# False-negative errors in next-generation sequencing contribute substantially to inconsistency of mutation databases

**DOI:** 10.1371/journal.pone.0222535

**Published:** 2019-09-12

**Authors:** Young-Ho Kim, Yura Song, Jong-Kwang Kim, Tae-Min Kim, Hye Won Sim, Hyung-Lae Kim, Hyonchol Jang, Young-Woo Kim, Kyeong-Man Hong

**Affiliations:** 1 Research Institute, National Cancer Center, Ilsan-ro, Ilsandong-gu, Goyang-si, Gyeonggi-do, Korea; 2 Department of Medical Informatics and Cancer Research Institute, College of Medicine, The Catholic University of Korea, Seoul, Korea; 3 Department of Biochemistry, College of Medicine, Ewha Womans University, Seoul, Korea; 4 Center for Gastric Cancer, National Cancer Center Hospital, Ilsan-ro, Ilsandong-gu, Goyang-si, Gyeonggi-do, Korea; Ohio State University Wexner Medical Center, UNITED STATES

## Abstract

**Background:**

More than 11,000 laboratories and companies developed their own next-generation sequencing (NGS) for screening and diagnosis of various diseases including cancer. Although inconsistencies of mutation calls as high as 43% in databases such as GDSC (Genomics of Drug Sensitivity in Cancer) and CCLE (Cancer Cell Line Encyclopedia) have been reported, not many studies on the reasons for the inconsistencies have been published. Methods: Targeted-NGS analysis of 151 genes in 35 cell lines common to GDSC and CCLE was performed, and the results were compared with those from GDSC and CCLE wherein whole-exome- or highly-multiplex NGS were employed.

**Results:**

In the comparison, GDSC and CCLE had a high rate (40–45%) of false-negative (FN) errors which would lead to high rate of inconsistent mutation calls, suggesting that highly-multiplex NGS may have high rate of FN errors. We also posited the possibility that targeted NGS, especially for the detection of low-level cancer cells in cancer tissues might suffer significant FN errors.

**Conclusion:**

FN errors may be the most important errors in NGS testing for cancer; their evaluation in laboratory-developed NGS tests is needed.

## Introduction

Whereas classical genetic tests measure limited base changes or structural changes in DNA, recently introduced next-generation sequencing (NGS) technology can examine millions of DNA variants at a time. According to the American Clinical Laboratory Association (ACLA), over 11,000 laboratories and companies in the USA have developed their own NGS tests [1]. Despite widespread adoption of NGS technology for the purposes of clinical diagnosis, high error rates on various NGS platforms has been reported (0.26–12.86%) [2]. In a study with 20,000 samples, the NGS false-positive rate was 1.3%, the authors suggesting that Sanger confirmation is required for NGS panel testing [3]. Some of the errors seem to be related to the NGS instrumentation itself, the SOLiD platform having shown an erroneous variant calling rate as high as 20–40% in a study [4]. Currently, in the USA, the clinical laboratories for NGS testing are overseen by the Centers for Medicare and Medicaid Services (CMS) according to the Clinical Laboratory Improvement Amendments (CLIA) regulations [5]. The Food and Drug Administration (FDA) has finalized a guidance document to accelerate the establishment of a regulatory approach for NGS testing [6,7], though the actual regulations are still debated. To establish any firm regulatory rules, further studies on the reproducibility or reliability of NGS results are needed.

For NGS analyses, target enrichment, amplification, sequencing processes, and bioinformatics analyses are involved, but the estimation of NGS errors have not been comprehensive [6,7]. Mutation calls from two cell-line databases including GDSC (Genomics of Drug Sensitivity in Cancer) and CCLE (Cancer Cell Line Encyclopedia) were constructed by whole-exome- or equivalently highly-multiplex NGS; inconsistent mutation calls have been reported to be as high as 43% [8], suggesting a possible high rate of error in NGS. Another recent report, however, denied the possibility of substantial NGS inconsistencies in mutation calls between GDSC and CCLE, arguing that not NGS errors but genetic evolution is the most important reason for inconsistency of mutation calls by NGS and for inconsistencies of drug responses in cell lines between GDSC and CCLE [9]. In order to investigate the reasons for discrepancies between the two databases or for errors in the highly-multiplex NGS, we performed targeted next-generation sequencing (T-NGS) on 151 genes in 35 cell lines common to GDSC and CCLE, and compared the results with those from CCLE and GDSC.

## Materials and methods

### Retrieval of mutation calls from GDSC and CCLE databases

The mutation calls for cancer cell lines were retrieved from the GDSC [10] and CCLE [11] databases. The GDSC mutation calls were down-loaded as an excel worksheet file [12] which had been constructed by whole-exome sequencing with hybrid capture for 1,047 cell lines. Additionally, the mutation calls for 1,650 genes using hybrid capture in 947 cell lines were downloaded as a Mutation Annotation Format (MAF) file from CCLE [13]. Later versions of the GDSC and CCLE data also were downloaded from the respective relevant sites [14,15].

### Mutation calls from T-NGS for 170 genes in 35 cell lines

For T-NGS, 35 cell lines present in both the GDSC and CCLE databases were selected (S1 Table). The 34 cell lines of NCI-60 cells, except for CALU6, were acquired from the National Cancer Institute (MTA No. 2702–09), as noted in our previous study [16]. CALU6 was purchased from Korean Cell Bank. The cells were cultured in RPMI-1640 culture media (Thermo Fisher Scientific Hyclone, Logan, UT, USA) containing 10% fetal bovine serum (Thermo Fisher Scientific Hyclone) at 37°C under 5% CO_2_. After the authentication of cell identities with short tandem repeat (STR) markers by the Omics Core Laboratory at the National Cancer Center, T-NGS was performed using the Axen Cancer Panel 2 (Macrogen, Korea), which can capture the exon sequences of 170 genes (S2 Table), though data for only 151 genes (S2 Table) were available each of the CCLE and GDSC databases. T-NGS was performed to achieve a median target coverage of 1000x. Basic data on the targeted sequencing with Axen Cancer Panel 2 is available in S3 Table.

Alignment of targeted sequencing reads with the reference sequence (hg19, UCSC genomes) was performed using the Burrows-Wheeler Aligner (BWA) [17] to produce SAM files from FASTQ files. Sequence quality was assessed by FastQC (https://www.bioinformatics.babraham.ac.uk/projects/fastqc/) based on the phred-scaled score with a cutoff value of 20. Duplicated reads were removed by Picard (http://broadinstitute.github.io/picard/). Local realignment and score recalibration were performed with the Genome Analysis Tool Kit (GATK) [17]. In the cases of mutation calls, GATK MuTect2 in the tumor-only mode was used for variant call format (VCF) file generation [18], and Oncotator (portals.broadinstitute.org/oncotator/) was used for annotation, which two processes resulted in the final MAF file. MAF files from our T-NGS in 35 cell lines are available at https://github.com/gensdei/CellineTargetedSeq.

### Selection of mutations among variation calls and definitions for possible true or false call rates

Among the variations in the T-NGS results, silent variants and those in the intronic or intergenic region were deselected first. Then, the exonic or splicing variation calls with a population AF of less than 1% based on databases from the 1000 Genomes Project (http://www.internationalgenome.org/1000-genomes-browsers/) and the UCSC genome were selected. Additionally, short in/del variants, which are frequently found in 35 cancer cells, also were removed (S4 Table), and the rest were used for further comparison analysis. For mutation calls from CCLE and GDSC, the variants showing population allele frequencies higher than 1% were removed.

The rate of true-positive or false-negative mutation calls can be estimated only when the true mutational status is known, but the data are not currently available. In the present study, we estimated the possible true-positive (P-TP), the possible false-positive (P-FP), and the possible false-negative (P-FN) rates, instead of the true-positive (TP), false-positive (FP), and false-negative (FN) rates, respectively, under the assumption that mutation calls with mutant allelic frequency (mAF) ≥ 10% (high mAF or hmAF) in our T-NGS were true-positive mutations. Therefore, the P-TP rate was calculated by the number of mutation calls in the databases divided by the number of hmAF calls in the T-NGS. P-FP rate was estimated by the number of CCLE-specific or GDSC-specific calls divided by the number of hmAF calls in the T-NGS. P-FN rate was estimated by the difference of the number of hmAF calls and that of CCLE-specific or GDSC-specific calls divided by the number of hmAF calls in the T-NGS. The inconsistency rate was calculated according to the total number of inconsistent mutation calls divided by the sum total of mutation calls.

### Validation of presence of mutation calls by PCR-sequencing

For validation of the presence of mutations in the cell lines, 10 genes (*ABL1*, *ALK*, *AR*, *BRD4*, *CDKN2A*, *ETV6*, *NOTCH4*, *PTCH1*, *RUNX1*, and *STK11*) were selected. Sanger sequencing was performed after PCR amplification with primers (S5 Table), employing the purified cell DNA from each cell line containing the specific mutation.

### Statistical analysis

Comparison of the mAFs between the two T-NGS was performed by linear regression. The Mann-Whitney test was used for determination of the significance of difference in the mutation read counts between the groups. Fisher’s exact test or the Chi-square test was used for determination of the significance in the difference of distribution between the P-TP and P-FN groups, in the difference of distribution of mutations in CpG-island regions between the groups, and in the difference of median read counts between the groups.

## Results

### Consistency of mutation calls from repeated T-NGS for 170 genes in 9 cell lines

To evaluate the reproducibility of our T-NGS, repeated targeted sequencings (designated TarSeq1 and TarSeq2 for the two T-NGS results, respectively) with two technical replicatess from the same DNA samples were performed for 9 cell lines (S1 Table). From the T-NGS results, totals of 688 and 603 mutation calls from TarSeq1 and TarSeq2, respectively, for 170 genes (S2 Table) in 35 cell lines (S1 Table) were obtained after removal of nonsense mutation calls, intronic or intergenic mutation calls, and all mutation calls with referenced SNP from the annotated data with Oncotator.

**Fig 1:**
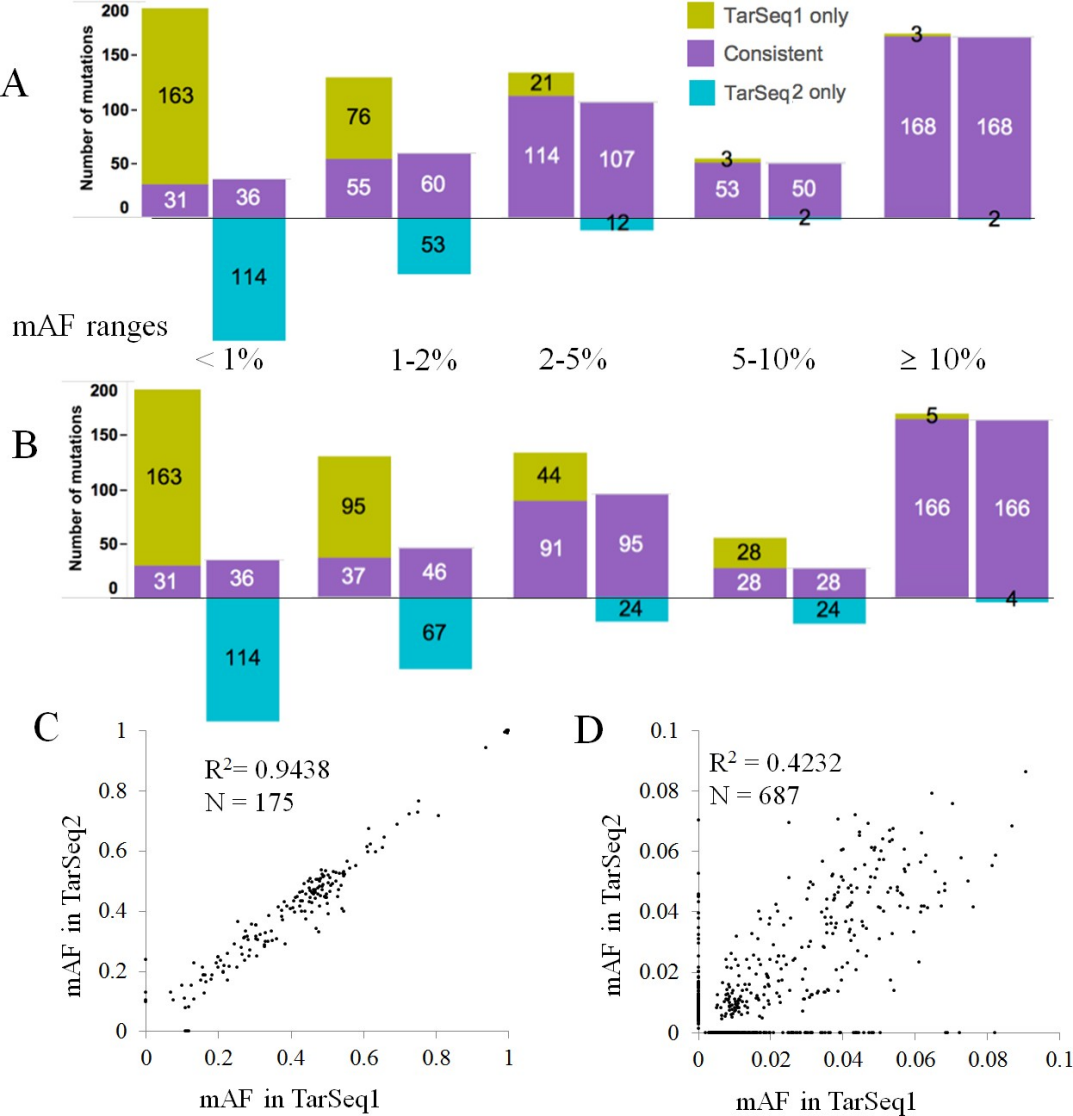
Dependence of consistency in mutation calls on cutoff level of mAF for repeated targeted sequencings. A. Consistency between first (TarSeq1) and second (TarSeq2) targeted sequencings for indicated mAF ranges {< 1%, 1% ≤ and < 2% (1–2%), 2% ≤ and < 5% (2–5%), 5% ≤ and < 10% (5–10%), and ≥ 10%}, when all mutation calls with any mAFs in either TarSeq1 or TarSeq2 are considered as true mutations. B. Consistency between two targeted sequencings, when mutation calls with mAF value ≥ 10% for both TarSeq1 and TarSeq2 are considered as true mutations. For both A and B, consistent calls between the two results are marked in purple, calls only from TarSeq1 (TarSeq1 only) in light green, and calls only from TarSeq2 (TarSeq2 only) in light blue; the left and right paired columns for each AF range are the data from TarSeq1 and TarSeq2, respectively; X-axis, mutant AF ranges; and Y-axis, number of mutation calls. C. Comparison of mutant AFs between two targeted sequencings results such as for hmAF mutation calls showing mAF ≥ 10% (R = 0.9438). D. Comparison of mAFs for calls showing mAF < 10% between two targeted sequencings (R = 0.4232). For C and D, X-axis is for TarSeq1 and Y-axis is for TarSeq2.

The consistency rate of mutation calls from the two T-NGS results was closely dependent on the mAF. When all of the mutation calls with any mAFs in either TarSeq1 or TarSeq2 were considered as true mutations, the consistency rates of mutation calls for mAF of < 1%, 1% ≤ and < 2%, 2% ≤ and < 5%, 5% ≤ and < 10%, and ≥ 10% were 19.5% (67/344), 47.1% (115/244), 87.0% (221/254), 95.3% (103/108), and 98.5% (336/341), respectively (Fig 1A). When the mutation calls with the mAF value of 10% for both TarSeq1 and TarSeq2 were considered as true mutations, the consistency rate was still high (97.4%, 332/341, (Fig 1B). However, when the lowest mAF value in each comparison group was employed as the cutoff value, the consistency rates for calls with mAFs of 5% ≤ and < 10%, 2% ≤ and < 5%, 1% ≤ and < 2%, and < 1%, were 51.9% (56/108), 73.2% (186/254), 33.9% (83/245), and 19.5% (67/344) (Fig 1B), respectively, indicating that only mutation calls with mAF ≥ 10% (hmAF calls) can be highly reproducible. Therefore, only hmAF mutation calls were further analyzed for comparison with the GDSC and CCLE databases in the present study.

In the comparison of the mAFs in the two T-NGS results, hmAF calls showed a high correlation between TarSeq1 and TarSeq2 (R^2^ = 0.9438, *P* < 0.0001, N = 175, Fig 1C) where the calls with read counts less than 20 (N = 4) were excluded. In contrast, the low-mAF (AF < 10%) calls showed relatively lower correlations between TarSeq1 and TarSeq2 (R^2^ = 0.4232, *P* < 0.0001, N = 687, Fig 1D), suggesting a higher chance of inconsistency in mutation calls with low mAFs.

### Mutation calls from T-NGS in 35 cell lines and their consistencies with those from GDSC and CCLE

For the evaluation of the true or false mutation call rates in GDSC or CCLE, targeted sequencing was performed for 151 genes common to both mutation databases in 35 cell lines (S1 and S2 Tables). In the T-NGS results, a total of 625 hmAF (mutant AF ≥ 10%) mutation calls (S6 Table) were obtained. Between the two independent targeted sequencings from 9 cell lines, hmAF mutation calls only from TarSeq1 data were employed for further analyses.

During the removal of SNPs from calls by our T-NGS, we found that 3 calls were found in more than 80% of cell lines (P1189fs in *MAP3K4* in 34 cells, L16fs in *NOTCH4* in 29 cells, and S941fs in MAP3K1 in 33 cells), but their population AF has not been reported. CCLE reported those 3 calls in 4 cells for P1189fs, in 4 cells for L16fs, and in 26 cells for S941fs, and GDSC reported S941fs in a cell, suggesting, after consideration of the high frequency of the calls in cells by our T-NGS, that these 3 calls may be germline SNPs. All of the cells wherein these 3 calls were identified in CCLE and GDSC were also identified by our T-NGS, suggesting that GDSC or CCLE have errors even in calling SNPs. To reduce errors in calling SNPs mutations, in the present study, we removed these calls from the list of mutation calls for further analyses, including others that were frequently found in our T-NGS but not in CCLE or GDSC (S4 Table).

**Fig 2:**
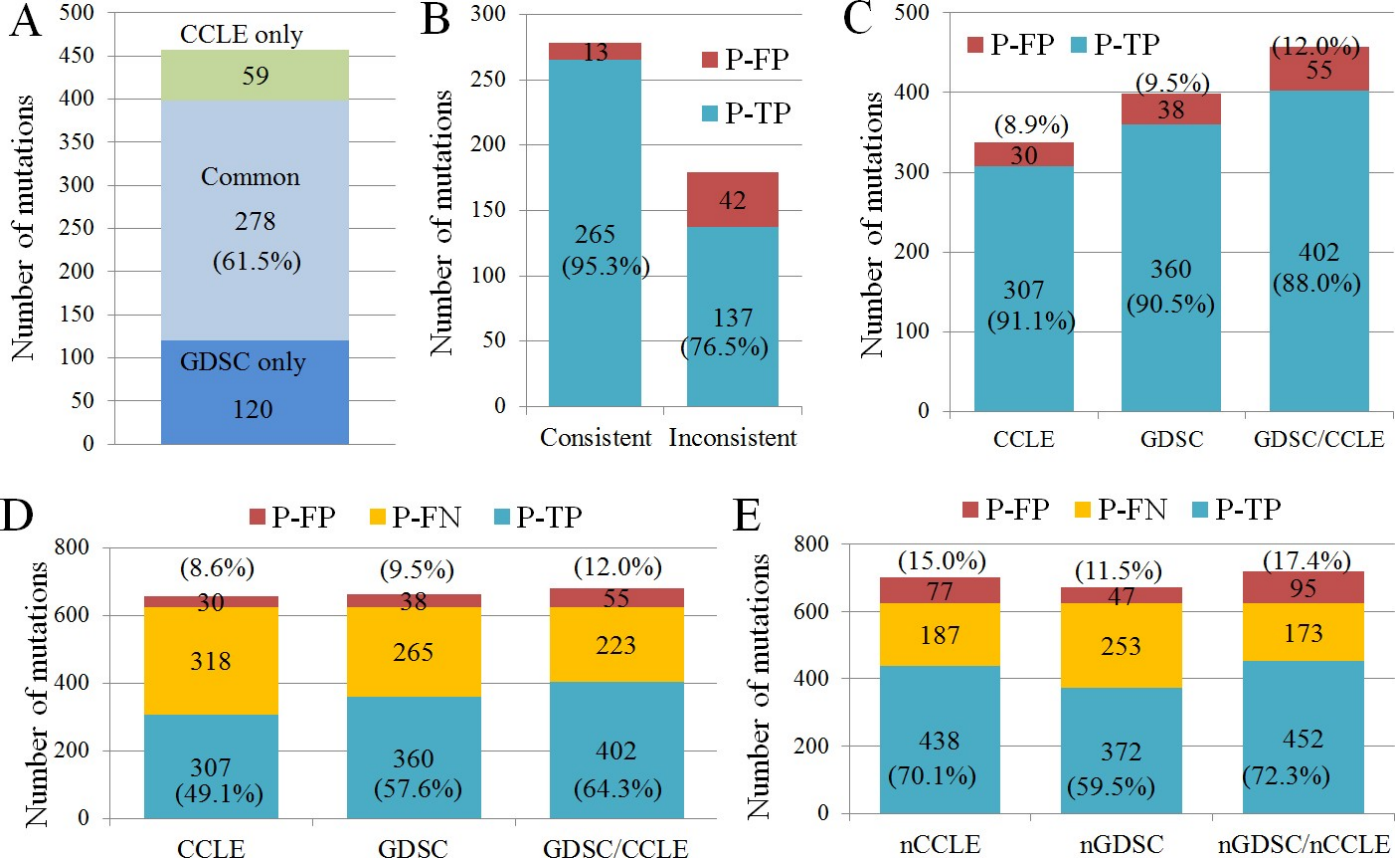
Estimation of error rates in mutation calls from CCLE and GDSC. A. Comparison of mutation calls between CCLE and GDSC for 151 genes in 35 cell lines. The consistency between GDSC and CCLE was 61.5%. B. Mutation detection rates in consistent and inconsistent mutation calls between GDSC and CCLE for 151 genes in 35 cell lines. Almost all of the consistent calls (“Consistent” on X-axis, 95.3%) and 76.5% of the inconsistent calls (“Inconsistent” on X-axis) between the two databases were positive (P-TP) in our T-NGS. C. P-FP call rates in GDSC and CCLE databases along with combined data from GDSC and CCLE databases (GDSC/CCLE). D. P-FP and P-FN mutation call rates in CCLE, GDSC, and combined data from GDSC and CCLE (GDSC/CCLE). E. P-FP and P-FN mutation call rates in later versions of CCLE (nCCLE) and GDSC (nGDSC), or in the combined data from later versions of GDSC and CCLE (nGDSC/nCCLE). P-TP, possibly true positive; P-FP, possibly false positive; P-FN, possibly false negative.

Newer versions of the GDSC and CCLE would contain updated mutation calls from various versions of NGS panels or from other sources including RNA sequencing results, and the comparison or estimation of mutation calls from various NGS experiments would not be appropriate. Therefore, for comparison of the T-NGS results with those from the databases, the older versions of the GDSC and CCLE databases were employed in the present study. A total of 470 mutation calls were found in the two databases (398 and 337 for GDSC and CCLE, each) for the 151 genes in 35 cell lines (S6 Table). The consistency rate between GDSC and CCLE was 61.5% (278/457) (Fig 2A), which is similar to that in a previous report [8].

Due to the unavailability of real true-positive (TP) or false-negative (FN) mutations in the cell lines, we calculated the P-TP or P-FN rates based on mutation calls from T-NGS, because hmAF mutation calls by our T-NGS were highly consistent in two T-NGS experiments (Fig 1B), along with the consideration that T-NGS is generally more sensitive and specific in target regions than are the whole-exome- and highly-multiplex NGS platforms that were employed for GDSC and CCLE. The P-TP rates were 95.3% (265/278) for consistent calls, and 76.5% (137/179) for inconsistent calls, between GDSC and CCLE (Fig 2B). This suggests that most of the inconsistent (76.5%) as well as consistent calls (95.3%) between the two databases are TP mutations, which is quite contrary to the general assumption that most inconsistent calls between the databases are FP calls.

### High rate of P-FN errors in GDSC and CCLE

As for the estimations of the P-TP rates of the calls in the databases, they were 91.1% (307/337) and 90.5% (360/398) for CCLE and GDSC, respectively (Fig 2C), suggesting that most calls by those databases are TP mutations. After combining the calls from the two databases (GDSC/CCLE), the P-TP rate was still high (88.0%, 402/457, Fig 2C).

The P-FP mutation rates were 8.9 (30/337), 9.5% (38/398), and 12.0% (55/457) in CCLE, GDSC, and GDSC/CCLE, respectively (Fig 2C). However, some of the P-FP mutations in CCLE (13/30) and GDSC (6/38) were identified as low-mAF mutations in our T-NGS. Therefore, the real P-FP rates, for which CCLE-only or GDSC-only calls showing low mAF in T-NGS were considered as P-TP calls and excluded from P-FP call count, would be about 5.0% (17/337), 8.0% (32/398), and 7.9% (36/457) in CCLE, GDSC, and GDSC/CCLE, respectively, suggesting that the rates of FP calls in the GDSC and CCLE databases may be relatively low.

For the estimation of the positive identification rates of TP mutations in CCLE and GDSC, the positive identification rates of P-TP mutations were calculated: those were only 49.1% (307/625) in CCLE and 57.6% (360/625) in GDSC (Fig 2D), suggesting that false negativity (42–51%) is a bigger problem than false positivity in whole-exome- or highly-multiplexed NGS, especially when the relatively low P-FP rate (4.7–8.0%) was considered. In addition, out of the 625 hmAF calls from our T-NGS, as many as 35.7% of the true mutations (223/625) could not be found in either GDSC or CCLE (Fig 2D), suggesting that the analyses employing two whole-exome- or equivalently highly-multiplex NGS platforms might not solve the major issue of FN in whole-exome NGS.

The GDSC and CCLE databases have been updated, more notably for the CCLE (in 2018). In the later CCLE version, the positive identification rate of P-TP mutations was greatly improved to 70.1% (438/625, nCCLE in Fig 2E), whereas the rate in the later GDSC version remained 59.5% (372/625, nGDSC in Fig 2E). Along with the increased identification rate of P-TP mutations, the P-FP rate also was increased, up to 15.0% (77/702) in the updated CCLE (nCCLE in Fig 2E). When the updated versions of GDSC and CCLE were combined (Fig 2E), the positive identification rate increased to 72.3% (452/720), but the P-FP rate also increased to 17.4% (95/547).

### FN errors significantly contribute to inconsistency between GDSC and CCLE

**Fig 3:**
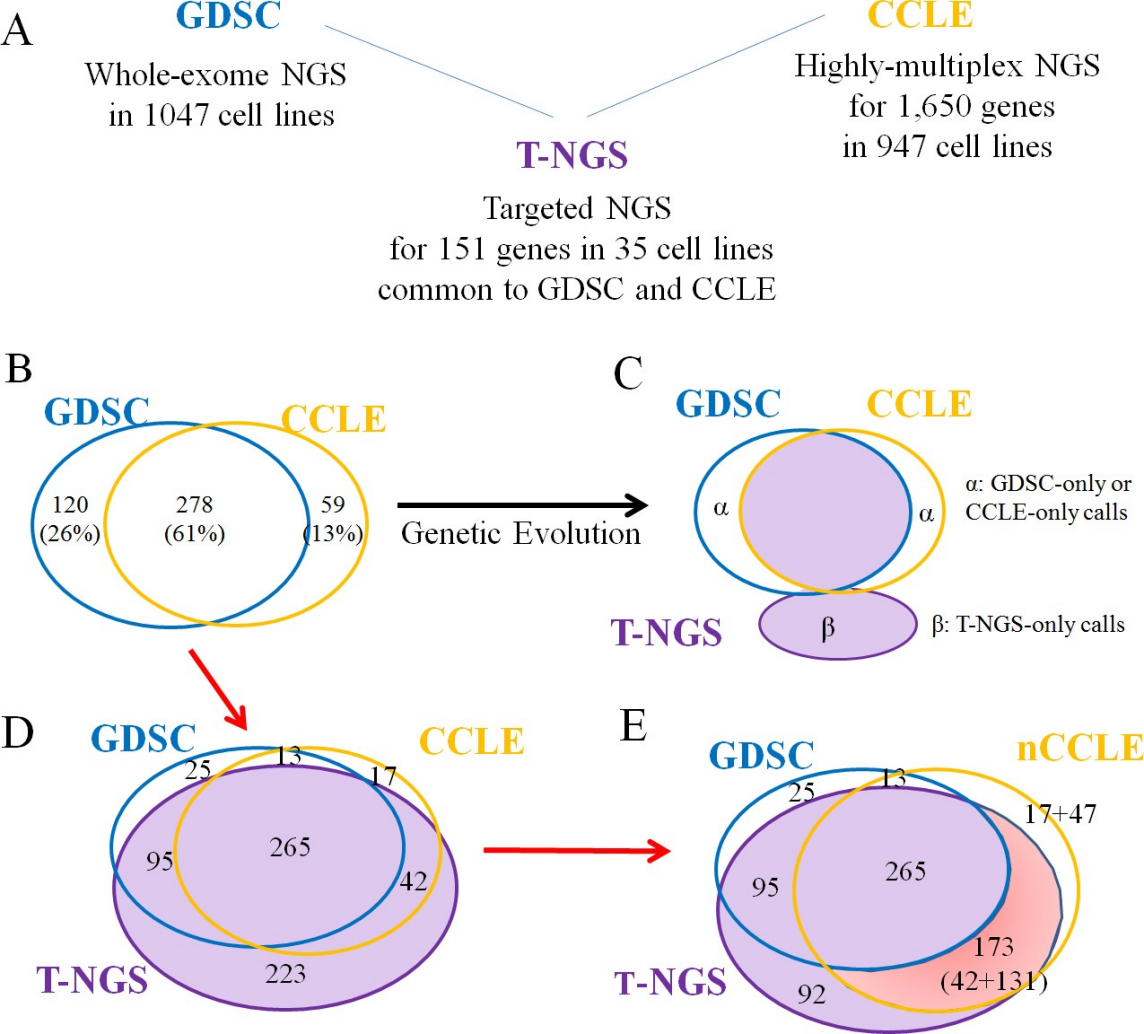
FN errors and inconsistent mutation calls between GDSC and CCLE. A. Comparison of results from GDSC, CCLE, and T-NGS. B. Inconsistent mutation calls between GDSC and CCLE for 151 genes in 35 cells. C. Expected mutational profiles from 3 independent laboratories, if genetic evolution is the major reason for the inconsistent mutation calls. If genetic evolution were the major factor for the difference between GDSC and CCLE, T-NGS would not contain calls specific to GDSC or CCLE (areas marked as α), but rather, additional calls specific to T-NGS (β). D. Inconsistent mutation calls among T-NGS, GDSC and CCLE. The number of calls found only in GDSC (25) or CCLE (17) was only about 10% of the T-NGS-only calls (223). In addition, 62% (111/179) of the inconsistent calls between GDSC and CCLE were found in our T-NGS. E. Consistency of T-NGS with later version of CCLE. In the later version of the CCLE database, 59% (131/223) of the T-NGS-only mutation calls in C were updated as newly identified mutations.

Even though inconsistent mutation calls and drug responses in cell-line databases such as GDSC and CCLE have been reported [8,19,20], a more recent report [9] insisted that genetic evolution in cell lines is an important reason for such inconsistency. To infer the possible reasons for the inconsistencies among databases, we compared the whole-mutation call data from GDSC, CCLE, and our T-NGS (Fig 3).

In the comparison of calls between GDSC and CCLE, 39.2% (179/457) were inconsistent (Fig 3A). In the comparison of the results from GDSC, CCLE, and our T-NGS, the expected mutational distribution would have been similar to the diagram shown in Fig 3B, if genetic evolution were the major factor behind the inconsistency between GDSC and CCLE, as suggested previously [9]: the diagram shows distinct laboratory-dependent mutations acquired by genetic evolution as well as distinct laboratory-independent common mutations originating from mother cancer cells.

If the genetic evolution were the major factor for the difference between GDSC and CCLE, T-NGS would not contain calls specific to GDSC or CCLE (marked α in Fig 3C). In our comparison of the calls, however, most of the calls specific to GDSC or CCLE were found in T-NGS: 97.2% (95/120) for GDSC 71.2% (41/59) for CCLE (Fig 3D). In the comparison, we also found many T-NGS-only calls (N = 223) that would be considered to be results of advanced genetic evolution (β in Fig 3C) if genetic evolution were the major factor. However, most of the T-NGS-only calls (58.7%, 131/223) among the mutation calls from T-NGS and CCLE were found in the later version of CCLE (nCCLE, Fig 3E), which contradicts the argument that genetic evolution is the major factor behind the differences in mutation calls between GDSC and CCLE [9].To confirm the presence of T-NGS-only mutation calls in cell lines, we performed PCR sequencing. Ten genes showing the highest T-NGS-only calls were selected for the analysis, in which genes a total of 54 mutation calls were found. Among the 54 calls, the presence of 29 mutations had already been shown in GDSC or the updated CCLE database (S6 Table). Therefore, PCR sequencing was performed for the remaining 25 mutations, and all were confirmed to be positive (S7 Table).

### Contribution of CpG-island regions to false negativity in NGS

**Fig 4:**
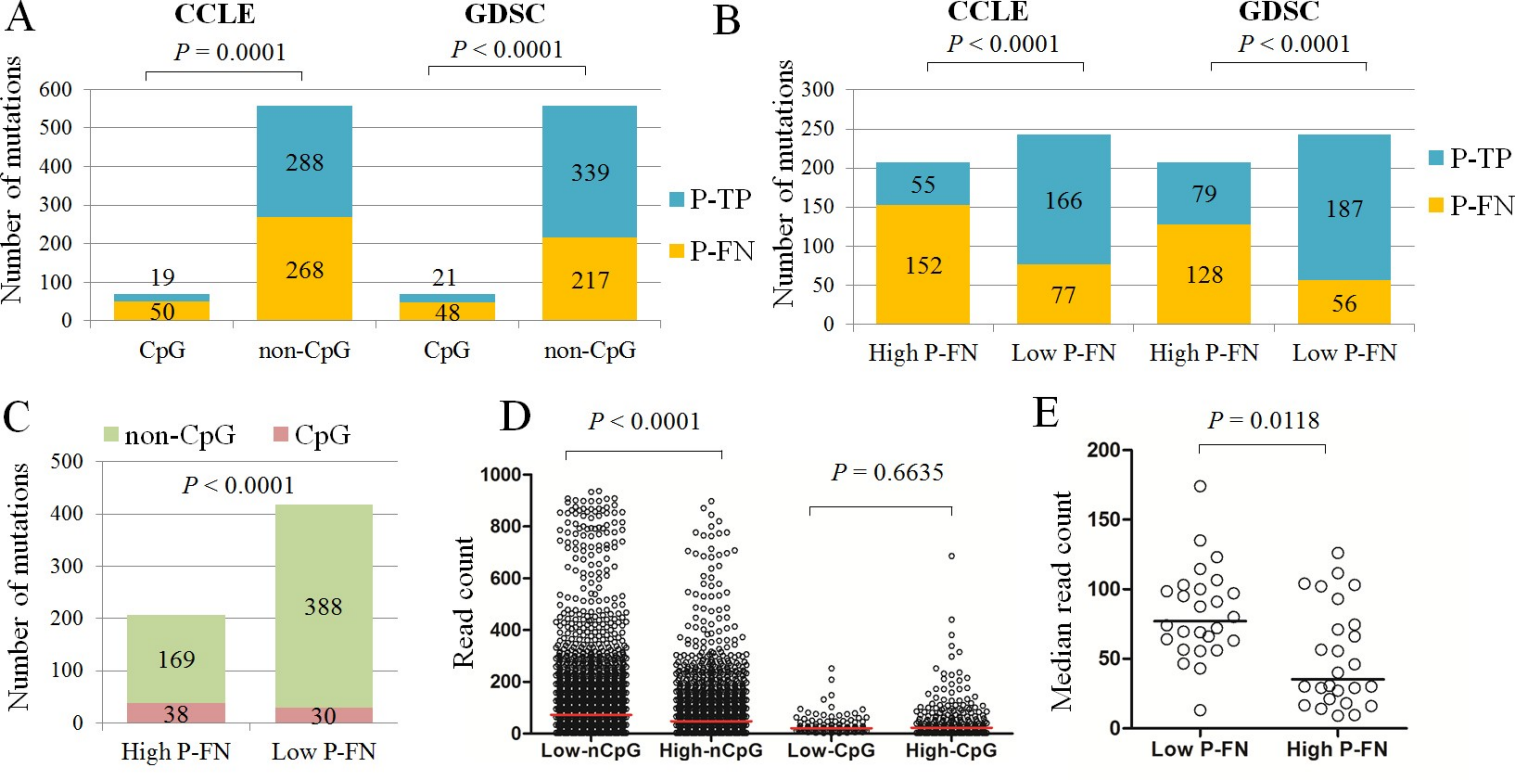
Various P-FN rates depends on genes or regions by whole-exome- or highly-multiplex-NGS analysis. A. Significantly higher P-FN calls in CpG mutation calls (CpG) than non-CpG mutation calls (non-CpG) for 151 genes in 35 cells from both CCLE and GDSC. However, the percentage of CpG mutations out of total P-FN calls was only 15.7% (50/318) in CCLE or 18.1% (48/265) in GDSC. B. Significantly higher P-FN rate in high P-FN group (High P-FN) than in low P-FN group (Low P-FN) for both CCLE and GDSC mutation calls. C. Higher rate of CpG mutation calls (CpG) in high P-FN group (High P-FN) than in low P-FN group (Low P-FN) for 151 genes in 35 cells. D. In an analysis of 54 genes in 947 cells from CCLE, significantly lower read counts of non-CpG mutation calls from the high P-FN group (High-nonCpG, N = 1,852) than from the low P-FN group (Low-nonCpG, N = 4,028) were observed, whereas there was no significant difference in the read counts of the CpG mutation calls between the high P-FN group (High-CpG, N = 267) and the low P-FN group (Low-CpG, N = 95). E. In an analysis of 54 genes in 947 cells from CCLE, a significantly lower median read count of mutations in the high P-FN group was observed after excluding the CpG mutations.

The contribution of CpG-island regions (CpG regions) in inconsistent mutation calls between GDSC and CCLE has already been reported [8]. In our estimation of the rates of mutation calls in CpG regions (CpG mutation calls) and those in non-CpG regions (non-CpG mutation calls) between the P-FN and P-TP calls from CCLE or GDSC, the importance of CpG-island regions was confirmed: In the analysis of all 625 hmAF mutations found in our T-NGS, the P-FN call rate was higher for CpG mutations than for non-CpG mutations in both CCLE and GDSC (*P* = 0.0001, for CCLE; *P* < 0.0001 for GDSC, Fig 4A), which suggests a significant contribution of CpG regions to FN-errors in NGS.

In contrast to the significant contribution of CpG regions to P-FN errors in whole-exome NGS, the percentage of CpG mutations out of the total P-FN calls was only 15.7% (50/318) in CCLE or 18.1% (48/265) in GDSC (Fig 4A), suggesting that CpG regions contribute only a small portion of FN errors in NGS.

### Identification rates of mutations by NGS depending on genes or regions

The P-FN call rate in CCLE were quite variable (0–100%) depending on various genes (Fig 4B), which fact suggests that FN rates may depend on specific genes. To analyze the correlation between specific genes and FN errors in the databases, we classified the 52 genes showing 5 or more mutation calls by T-NGS in 35 cell lines into low P-FN gene (P-FN < 60%, N = 26) high P-FN gene (P-FN ≥ 60%, N = 26) groups depending on the P-FN rates of genes in CCLE (Table 1).

**Table 1:** List of genes for high and low P-FN groups.

High P-FN Group*	CCLE^b^	T-NGS^c^	P-FN Rate^a^	Low P-FN Group**	CCLE^b^	T-NGS^c^	P-FN Rate^a^
**BRD4**	0	5	1.00	**KRAS**	10	10	0.00
**NOTCH4**	0	5	1.00	**PIK3CA**	10	10	0.00
**RUNX1**	0	5	1.00	**PTEN**	8	9	0.11
**PTCH1**	1	8	0.88	**MLH1**	5	6	0.17
**ABL1**	1	6	0.83	**BRAF**	9	11	0.18
**AR**	1	5	0.80	**CHEK2**	4	5	0.20
**ALK**	1	5	0.80	**MAP3K1**	4	5	0.20
**CDKN2A**	1	5	0.80	**NF1**	11	14	0.21
**ETV6**	1	5	0.80	**BRCA2**	7	9	0.22
**STK11**	1	5	0.80	**BRD3**	7	9	0.22
**RICTOR**	2	9	0.78	**CREBBP**	5	7	0.29
**ATR**	2	9	0.78	**ERBB3**	5	7	0.29
**NOTCH3**	4	17	0.76	**PDGFRA**	4	6	0.33
**JAK3**	2	7	0.71	**ATM**	8	12	0.33
**TSC2**	3	10	0.70	**PIK3CB**	4	6	0.33
**NOTCH1**	4	13	0.69	**RET**	4	6	0.33
**NOTCH2**	4	13	0.69	**APC**	13	21	0.38
**MSH6**	4	13	0.69	**SYK**	3	5	0.40
**FLT4**	3	9	0.67	**RAD50**	3	5	0.40
**NTRK1**	2	6	0.67	**FLT1**	3	5	0.40
**MAP3K4**	2	6	0.67	**KDR**	4	7	0.43
**FLT3**	4	11	0.64	**AXL**	5	9	0.44
**CSF1R**	2	5	0.60	**TP53**	17	32	0.47
**MET**	2	5	0.60	**ROS1**	4	8	0.50
**SMARCA4**	6	15	0.60	**GNAS**	6	12	0.50
**MSH2**	2	5	0.60	**BRCA1**	3	7	0.57

Each gene among genes with 5 or more mutation calls in T-NGS is allocated to high P-FN group (*, P-FN ≥ 60%) or low P-FN group (**, P-FN < 60%), based on the P-FN rates (^a^) for each gene in CCLE.

The numbers of mutations identified by CCLE (^b^) and T-NGS (^c^) are indicated.

Between the low and high P-FN groups, the P-FN rate was significantly higher in the high P-FN group in CCLE (*P* < 0.0001, Fig 4B), as expected. If the higher P-FN rate in the high P-FN group had been observed also in GDSC, the FN errors in the high P-FN group would not be unique to CCLE but rather, a common phenomenon for whole-exome- or highly-multiplexed-NGS platforms. In fact, the significantly higher P-FN rate in the high P-FN group was also observed in GDSC (*P* < 0.0001, Fig 4B), suggesting that certain common genes or regions can be significantly related to FN errors in highly-multiplexed NGS platforms.

In the analysis of the significance of CpG regions in the low or high P-FN group, significantly more fraction of CpG mutations were found in the latter than in the former group (Fig 4C, *P* < 0.0001), indicating that CpG regions are significantly correlated with the high P-FN group.

For further insights into mutation calls in the high P-FN group, the read counts of the mutations for 52 genes of the low (N = 4122) and high (N = 2118) P-FN groups in 947 cell lines were retrieved from CCLE and compared (Fig 4E and 4F). Although the read counts of CpG mutation calls were low in both the low and high P-FN groups (Fig 4D), they were not significantly different between those groups (*P* = 0.6635, Fig 4D). However, in the non-CpG regions, the read counts of non-CpG mutation calls were significantly lower in the high P-FN group than in the low P-FN group (*P* < 0.0001, Fig 4D). In addition, the median read count of non-CpG mutations from genes was significantly lower in the high P-FN group than in the low P-FN group (Fig 4E, *P* = 0.0118). These results suggest that lower read counts due to other-than CpG mutation calls are the major causal factor behind FN errors in NGS.

## Discussion

We performed T-NGS to investigate the reasons for inconsistencies in mutation calls between GDSC and CCLE, which databases had been constructed by whole-exome- or equivalently highly-multiplex NGS. In our sequencing-NGS analysis for 151 genes in 35 cell lines, the P-FN rates in GDSC or CCLE were as high as 42–51%, whereas the P-FP rates were only 5.0–8.0%, suggesting that FN errors may be the most important reason for the discrepancy between databases and for errors in highly-multiplex-NGS analyses.

Whole-exome NGS has been widely adopted for clinical application in the fields of precision medicine and personalized neo-antigen vaccine therapy [21,22]. Large cell-line mutation databases such as GDSC and CCLE have been constructed by employing whole-exome- or equivalently highly-multiplex NGS [10,11]. However, inconsistencies between GDSC and CCLE have been found [4,8,19]. To investigate the possible reasons for such inconsistency, the present study performed T-NGS. We assumed that T-NGS can reduce errors incurred by whole-exome NGS and yield more reliable information on mutational status in cell lines. In our analysis of two consecutive targeted sequencings, the mutation calls with mutant AF >10% (hmAF calls) in targeted sequencing were highly reproducible, and as such, were considered to be true-positive mutations. In the comparison of our T-NGS results with the mutation calls in GDSC or CCLE, we found that the databases contained 49–58% of P-TP mutations, in contrast to the relatively low P-FP rate (7.9%). In addition, most (62%) of the inconsistent mutation calls between GDSC and CCLE were identified in our T-NGS, whereas 7.9% of mutations in the databases were not identified in our T-NGS, and 59% of the mutation calls found only in our T-NGS but not in CCLE were found in the updated version of CCLE. Therefore, we concluded that the inconsistent mutation calls between GDSC and CCLE are related to FN errors.

Inconsistency of mutation calls between cell-line databases might be related to genetic evolution, as suggested recently [9]. In the comparison of our T-NGS results with those from GDSC and CCLE, we found 223 T-NGS-only mutation calls, 25 GDSC-only calls, and 17 CCLE-only calls (Fig 3C), all of which calls may be related to the genetic evolution of the cell lines. If genetic evolution is the primary reason for the discrepancy, the discrepancies would be patterns with core-consistent or laboratory-independent calls for all three databases, along with laboratory-dependent calls generated by genetic evolution in each laboratory with only a few calls being consistent in only two of the three databases. In our comparison, however, the discrepancy pattern was not what we expected: many calls were consistent in two out of the three databases, suggesting that genetic evolution is not the major factor behind the discrepancy. In addition, most of inconsistent mutation calls (62%, 111/179) between GDSC and CCLE were identified in our T-NGS, and there were few GDSC-only or CCLE-only mutations. Furthermore, 59% (131/223) of the T-NGS-only calls were reported in the later version of CCLE. These results suggest that the discrepancy of mutation calls between GDSC and CCLE might not originate from genetic evolution but rather from FN errors.

Whereas NGS has been often employed for clinical diagnosis, many reports have noted significant errors even in T-NGS [3,4,8] as well as a necessity for regulations on NGS testing [5,7,23]. Because the present study’s results indicate a high rate of FN errors in highly-multiplex NGS, repeating NGS experiments and/or reducing the cutoff level of mutant AF might be necessary to reduce the number of FN errors. However, our results indicated that the combination of GDSC and CCLE data did not greatly improve the positive identification rate of P-TP mutations (from 40~58% to 64%), and that the P-FP rate was only slightly increased or remained steady (from 5.0~8.0 to 7.9%). Reducing the cutoff level of mutant AF did not reduce the errors, either, because the inconsistency rate increased dramatically, from 2.6% with the cutoff value of mAF ≥ 10% to 67% with that between 1 and 2%. Therefore, our results suggest that simply repeating NGS experiments or changing the parameters for the analysis of NGS results might not be an effective way to reduce FN errors.

As most NGS tests employed in clinics currently are T-NGS [23], an important question would be if T-NGS tests are free of major FN-error issues. We showed in the present study that, in two consecutive T-NGS experiments, T-NGS can also encounter reproducibility problems. In fact, the consistency in the two NGS experiments was quite dependent on the cutoff level of mAF: the consistency was as high as 97–98% when the calls with mAF ≥ 10% were compared, but only 15–25% when the ones with mAF < 1% were compared. Because inconsistency can be related to FN errors, the application of a low mAF cutoff value in NGS data analysis should lead to a high FN rate in T-NGS analyses. Especially, the application of NGS to low-percentage cancer tissues might increase the number of FN errors if a lower mAF cutoff value is employed. The potential problems incurred by FN errors in T-NGS have already been raised in guidelines for validation of NGS-based oncology panels [23]. The present study compared the results only from hybrid-capture-based NGS for target enrichment in T-NGS methods. However, amplification-based NGS has already been known to be vulnerable to FN errors [23], suggesting that FN errors might be one of the most important errors in T-NGS, regardless of target-enrichment methods.

For the sensitive detection of rare mutants by NGS, Safe-Sequencing System (Safe-SeqS) [24] and barcode-free NGS validation method [25] have been developed. However, these methods were designed to reduce false positive errors, and the problem of false negative errors may not be solved. Therefore, careful evaluation of possible FN errors by multiplex-NGS tests even with the adoption of Safe-SeqS method or barcode-free NGS validation method may be needed, especially when extremely low mAF is employed for the analyses.

The present study’s overall results suggest that false negativity in NGS is a major factor behind the errors or discrepancies of mutation calls in GDSC and CCLE, though the reasons for the false negativity were not elucidated. CpG regions have been reported to be an important reason for discrepancies between GDSC and CCLE [8]. Consistently, the present study also showed that CpG regions were significantly related to higher P-FN rate of mutation calls, which finding indicates that CpG regions might be a significant factor behind inconsistency and/or FN errors in NGS analysis. However, the mutations in the CpG regions were only 16–18% of the P-FN calls in GDSC and CCLE, suggesting the possibility of another major causative factor for FN errors in NGS. The present study showed that specific genes in the high P-FN group may be prone to FN errors, because the P-FN rate was significantly higher in the high than in low P-FN group for both GDSC and CCLE. However, the reasons for the high P-FN group have not been clearly elucidated, except for the significantly lower read count of mutation calls. Further investigation of the possible reasons for the high P-FN group is needed to reduce FN errors in NGS.

## Conclusion

FN errors may be the most important reason for the errors or inconsistencies in highly-multiplex-NGS tests for mutation detection in cancer. Also in T-NGS tests, FN errors may be one of the most important factors for the analysis of cancer tissues with low-percentage cancer cells designed for extremely high-sensitivity detection of mutations.

## Supporting information

S1 Table List of cells analyzed in targeted NGS. (XLSX)

S2 Table List of genes analyzed in targeted NGS. (XLSX)

S3 Table The basic information of the targeted sequencing with Axen Cancer Panel 2. (XLSX)

S4 Table List of calls which are found frequently in targeted NGS and are considered as SNPs. (XLSX)

S5 Table Primers for the confirmation of P-FN calls. (XLSX)

S6 Table Mutations for 151 genes in 35 cancer cell lines. (XLSX)

S7 Table P-FN mutations which were confirmed from the GDSC or updated CCLE databases or by Sanger sequencing. (XLSX)

## Abbreviations

NGS: next-generation sequencing
WE-NGS: whole-exome-next-generation sequencing
TP: true positive
P-TP: possibly true positive
FN: false negative
P-FN: possibly false negative
FP: false positive
P-FP: possibly false positive
AF: allelic frequency
mAF: mutant allelic frequency
hmAF: mAF ≥ 10%
Safe-SeqS: Safe-Sequencing system
poorly AG: poorly analyzed gene
well AG: well analyzed gene
